# Distinct Effects of Brain Activation Using tDCS and Observational Practice: Implications for Motor Rehabilitation

**DOI:** 10.3390/brainsci14020175

**Published:** 2024-02-13

**Authors:** Julianne McLeod, Anuj Chavan, Harvey Lee, Sahar Sattari, Simrut Kurry, Miku Wake, Zia Janmohamed, Nicola Jane Hodges, Naznin Virji-Babul

**Affiliations:** 1 Rehabilitation Science, Faculty of Medicine, University of British Columbia, Vancouver, BC V6T 1Z3, Canada; jdmcleod@student.ubc.ca; 2 Electronics and Telecommunication Engineering, Sardar Patel Institute of Technology, Mumbai 400058, India; anuj.chavan@spit.ac.in; 3 Schulich School of Medicine & Dentistry, Western University, London, ON N6A 5C1, Canada; hlee2025@meds.uwo.ca; 4 Biomedical Engineering, Faculty of Applied Science and Faculty of Medicine, University of British Columbia, Vancouver, BC V6T 2B9, Canada; ssattari@student.ubc.ca; 5 Neuroscience, Faculty of Science, University of British Columbia, Vancouver, BC V6T 1Z3, Canada; simrut.kurry@gmail.com (S.K.); mikuwake@student.ubc.ca (M.W.); 6 Neuroscience, Faculty of Science, McGill University, Montreal, QC H3A 2B4, Canada; zia.janmohamed@mail.mcgill.ca; 7 School of Kinesiology, Faculty of Education, University of British Columbia, Vancouver, BC V6T 1Z1, Canada; nicola.hodges@ubc.ca; 8 Physical Therapy, Faculty of Medicine, University of British Columbia, Vancouver, BC V6T 1Z3, Canada; 9 Djavad Mowafaghian Centre for Brain Health, Vancouver, BC V6T 1Z3, Canada

**Keywords:** transcranial direct-current stimulation, effective connectivity, electroencephalogram, action observation, observational learning, motor skill acquisition

## Abstract

Complex motor skills can be acquired while observing a model without physical practice. Transcranial direct-current stimulation (tDCS) applied to the primary motor cortex (M1) also facilitates motor learning. However, the effectiveness of observational practice for bimanual coordination skills is debated. We compared the behavioural and brain causal connectivity patterns following three interventions: primary motor cortex stimulation (M1-tDCS), action-observation (AO) and a combined group (AO+M1-tDCS) when acquiring a bimanual, two-ball juggling skill. Thirty healthy young adults with no juggling experience were randomly assigned to either video observation of a skilled juggler, anodal M1-tDCS or video observation combined with M1-tDCS. Thirty trials of juggling were performed and scored after the intervention. Resting-state EEG data were collected before and after the intervention. Information flow rate was applied to EEG source data to measure causal connectivity. The two observation groups were more accurate than the tDCS alone group. In the AO condition, there was strong information exchange from (L) parietal to (R) parietal regions, strong bidirectional information exchange between (R) parietal and (R) occipital regions and an extensive network of activity that was (L) lateralized. The M1-tDCS condition was characterized by bilateral long-range connections with the strongest information exchange from the (R) occipital region to the (R) temporal and (L) occipital regions. AO+M1-tDCS induced strong bidirectional information exchange in occipital and temporal regions in both hemispheres. Uniquely, it was the only condition that was characterized by information exchange between the (R) frontal and central regions. This study provides new results about the distinct network dynamics of stimulating the brain for skill acquisition, providing insights for motor rehabilitation.

## 1. Introduction

Motor learning can be facilitated without physical practice by repeatedly observing the performance of a motor skill, referred to as observational learning [1,2,3]. Observational learning of motor skills is thought to involve a complex interaction within distributed brain networks involving motor as well as non-motor regions. These regions include the inferior parietal cortex (IPC), superior temporal sulcus (STS), inferior and superior frontal cortices and occipito-temporal regions, with mirror-like properties that respond both to the execution and observation of actions (e.g., [4,5,6]; see [3] for a recent review).

Much of the research in observational learning is on the adaptation or adjustment of actions that can already be performed, such as adaptation of throwing actions to a target, pressing keys to memorize a sequence, or tracking tasks. In some of our own research involving repeated observation of a joystick tracking task, observational practice induced neurophysiological changes as indexed by mu suppression at central sites, providing further evidence for motor-based processes of the action observation network (AON) being active during observational practice. However, we did not find that these motor-related processes were related to behavioural measures of learning [7]. In addition to the need to establish direct links between measures of brain activity and behavioural outcomes, little is known about the brain processes underpinning observational learning of new actions when the observer does not have an existing motor skill set to perform what they are watching. This is of particular importance when considering learning of new actions or re-learning actions following brain injury.

Transcranial direct-current stimulation (tDCS) is a non-invasive technique of neuromodulation via application of a weak current through the scalp. It is inexpensive, easy to use, and, more importantly, shows great promise to modify cortical excitability [8,9]. Due to its simplicity and portability, the effects of tDCS have been investigated in both healthy adults and in a range of neurocognitive disorders, such as major depressive disorder, bipolar disorder, Alzheimer’s disease [10,11], and traumatic brain injury [12] with varying degrees of success.

Over the past decade, there have been numerous reports evaluating the effects of tDCS on human motor performance and learning. Increasing the excitability of the primary motor cortex (M1) with tDCS during motor practice has been shown to improve motor performance for a range of skills such as the sequential finger tapping task [13,14], serial response reaction time tests [15], and visuomotor tracking [16]. These effects have primarily been reported for unimanual tasks (see [17] for a review), with few-to-none evaluating the uncoupled effects of tDCS. Pixa and Pollock [17] suggest that tDCS has the potential to enhance bimanual motor performance, yet there are a limited number of studies involving such skills and stimulation.

Effective connectivity (EC) provides a measure of the influence (direct or indirect) that one brain region exerts over another [18] and identifies causal, directionally dependent interactions between different brain regions. Little is known about the effects of tDCS at this network level, particularly when tDCS is applied over the motor network. Calzolari et al. [19] evaluated resting-state effective connectivity across the motor network after applying tDCS over M1 or the cerebellum in the same cohort on separate days. They reported changes beyond the targeted regions that were stimulated, including between the cortex, thalamus and cerebellum. However, they focused their analysis on the resting-state activity of the brain without using a motor learning task. A key question that arises from their work, and observational learning research in general, is: how is causal connectivity between brain regions modulated during action observation as compared with the application of M1-tDCS alone and the combination of action observation and tDCS when acquiring a novel bimanual task? In all cases, there is expected to be activity in motor-related regions through direct or indirect stimulation of the motor cortex and surrounding regions.

We investigated the patterns and statistics of effective connectivity using a data-driven effective brain connectivity measure that is based on the concept of information flow rate applied to electroencephalogram (EEG) signals (see [20]). The information flow rate was developed by Liang using the concept of information entropy and the theory of dynamical systems [21]. Information entropy is a measure of the information contained in a given signal (e.g., time series) and quantifies the changes in the information content of a time series (and hence, the temporal evolution of the brain region from which the signal is acquired) as a result of the interactions with other brain regions and the stochastic forces. The information flow rate measures the directional transfer of information between time series at different locations and, therefore, between different brain regions. A high information rate from region A to region B suggests that a large amount of information is transferred from A to B per second. Given a collection of source locations in the brain, the information flow rate can identify which sources transmit and which receive information, thus leading to a network of brain connections. Since the derived connectivity is directional, the information flow rate provides a robust method for detecting causal links in the brain.

The aim of this study was to use information flow rate to measure effective connectivity following AO, M1-tDCS and AO+M1-tDCS in three groups of healthy individuals. Such measures would allow us to compare the brain activity associated with direct and “indirect” stimulation (through passive observation) of motor-related brain areas, detailing the direction and spatial patterns of information flow. We were also interested in examining the outcomes of skill acquisition of a novel bimanual task resulting from the pairing of AO and M1-tDCS separately. We chose juggling as the novel bimanual coordination task, which requires simultaneous control and coordination of multiple movements [22].

Given the limited previous literature related to effective connectivity on observational practice and tDCS on novel bimanual tasks, our preliminary hypothesis extended from past research on unimanual tasks. We predicted that: (a) AO would be associated with activity in bilateral motor regions embedded within connections of the frontal-temporal-parietal action observation network, (b) M1-tDCS would be associated with a dominant nexus of information flow arising primarily from the (L) primary motor region with bidirectional information exchange over widespread cortical connections and (c) combining AO and M1-tDCS would be associated with connections in the action observation network as well as the (L) primary motor region.

## 2. Materials and Methods

### 2.1. Participants

Thirty participants with no juggling experience from the University of British Columbia were recruited for a study on the effects of tDCS and AO. Participants were randomly allocated to one of three intervention groups: action observation (AO), primary motor cortex stimulation (M1-tDCS), or combined action observation and tDCS (AO+M1-tDCS) (mean age: 22 ± 3 yr; 21 F; 9 M). In a previous study involving observational effects associated with repeated watching of a juggling action [23], there were moderate-to-strong effects associated with observational practice on overall juggling form scores (the same measure as we adopted here). Based on a mixed between and repeated-measures design, with a medium-large effect size of *f* = 0.42 (with 2 repeated measures; 3 groups), the sample size estimation was *N* = 27 (alpha = 0.05, power = 0.80).

Inclusion criteria included right-handed (based on the Edinburgh Handedness Inventory, [24]), normal or corrected-to-normal vision, no history of neurological or psychiatric disorders nor any contra-indications for tDCS (i.e., history of seizures or an intracranial implant). Individuals self-reported having no previous juggling experience. This study was approved by the University of British Columbia Behavioural Research Ethics Board-B (H17-03361). Consent was obtained from all participants before enrolment.

### 2.2. Study Design

**Figure 1:**
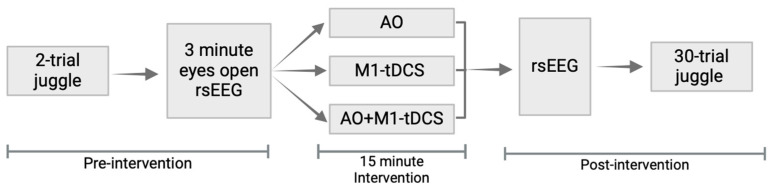
Experimental procedure. All participants performed a 2-trial juggle followed by 3 min eyes open resting-state EEG (rsEEG). Participants were randomized to one of three interventions: action observation (AO), tDCS stimulation (M1-tDCS), or action observation and tDCS stimulation (AO+M1-tDCS). Individuals in the AO group watched a video of a skilled juggler for 15 min. Individuals in the tDCS group received 15 min of weak direct current (2 mA) of anodal stimulation over the primary motor cortex (M1). Individuals in the AO+M1-tDCS group watched the skilled juggler video while simultaneously receiving 15 min of 2 mA anodal stimulation over M1.

The chronology of this study is outlined in Figure 1. All participants completed two, pre-intervention juggling trials, which were video-taped and saved for later analysis. A three-minute, eyes-open baseline EEG session was then recorded, after which participants were randomly assigned to one of three fifteen-minute intervention groups: (1) video observation of a skilled juggler/“AO group,” (2) M1-tDCS with no video observation/“M1-tDCS group” or (3) video observation and M1-tDCS “AO+M1-tDCS”. There was then a second three-minute, eyes-open resting-state post-intervention EEG period, immediately followed by thirty practice attempts at the two-ball juggling task, which were video-taped for analysis.

### 2.3. Juggling Task

Effects of the practice intervention were investigated using a two-handed “exchange” juggling task with two balls [25]. One juggling trial consisted of two throws and two catches, and participants were instructed that the balls had to cross in the air and that they had to toss the balls to the opposite hand. The two juggling balls were identical in size and weight. Participants were seated while attempting to juggle. A performance analysis was subsequently conducted on the video recordings according to previous scoring criteria [23]. Performance was scored using three criteria: hand release asymmetry, ball height symmetry, and whether one or two balls were caught. Each of the three criteria received a score ranging from 0 (symmetrical release, hand-over, low height, no balls caught) to 2 (asymmetrical release, approximately symmetrical peaks and both balls caught). The scores for each criterion were summed to obtain a total score for each juggle, with six denoting a perfect score. Two raters independently scored a subset of the data and agreed on the performance criteria using a standard process of consensus scoring. Pre-intervention juggling accuracy was assessed in a 2-trial juggle to limit motor learning due to practice.

### 2.4. Interventions

#### 2.4.1. Video Observation (AO)

Participants who engaged in video observation watched a 15 min video of a skilled juggler performing the two-ball juggling task from a 3rd-person perspective. Participants were not permitted to imitate the juggler while watching the video.

#### 2.4.2. M1-tDCS

Participants who received tDCS had 15 min (2 mA) of anodal stimulation (Starstim Neuroelectrics, Barcelona, Spain) over the left primary motor cortex (M1) according to the international 10–20 EEG system. Participants kept their eyes open during the stimulation.

### 2.5. EEG Recording and Preprocessing

Resting-state brain activity was recorded with electroencephalography (EEG) using a 64-channel HydroGel Geodesic Sensor Net at a 500 Hz sampling rate. Scalp electrode impedance values were confirmed to be below 50 k before recording. All recorded signals were referenced to Cz. Raw EEG data were preprocessed using EEGLAB (v2021.1) in MatLab (v2022b). Each subject’s EEG time series was re-referenced to the average, down-sampled to 250 Hz, and notch filtered at 60 Hz. We then applied a low-pass filter at 50 Hz and a high-pass filter at 0.5 Hz. Independent Component Analysis (ICA) was used to identify and remove any non-brain artifacts. Clean EEG sensor-level data was converted to source space using Brainstorm in MatLab [26]. An inverse modelling method of minimum norm estimate (MNE) with sLORETA was used. The ICBM152 template was used as the head model. The source space solution was projected to ten regions of interest (ROIs) based on the Deskan-Killiany cortical atlas.

### 2.6. Effective Connectivity

A full description of the information flow rate for application in the analysis of EEG source-reconstructed signals is provided in [20,21]. Below, we provide a summary of the methodology:

The information flow rate measures the rate of information transfer from the time series *i* to the time series *j* and can be expressed using the above definitions as follows [27]:

(1) $$T_{i\to j}=\frac{\hat{r_{ı,j}\;}}{1-\hat{r_{i,j}^{2}}}\left(\hat{r_{i,dj}}-\hat{r_{i,j}}\hat{r_{j,dj}}\right),$$

(2) $$for\;i,j=1,\ldots ,N_{s},\;i\neq j$$

We refer to *pi* as the transmitter series and to *pj* as the receiver series with respect to *Ti→j*. A positive (negative) rate of information flow from *i→j* (*Ti→j*) indicates that the interaction between the two series leads to an increase (decrease) in the entropy of the series *pj*. It also signifies that the receiver series becomes more (less) unpredictable due to its interaction with the transmitter series. The predictability of each time series is negatively correlated with the entropy.

The information flow rate *Ti→j* is a measure of the information flow from series pi to series *pj*, but it gives no indication of whether the impact of *pi* on the predictability of *pj* is significant. Quantifying the latter requires knowing the relative impact of the entropy transferred to the receiver from the transmitter series, compared to the total entropy rate of change due to all the influences acting on the receiver. The latter (hereafter referred to as the normalization factor for the information flow rate from *pi* to *pj* and denoted as *Zi→j*) can be computed [20,21]. The relative impact of the transmitter series on the receiver series is then given by the normalized information flow rate from the transmitter *pi* to the receiver *pj: τi→j = Ti→j/Zi→j,* which measures the percentage of the total entropy rate of change for *pj* that is due to its interaction with *pi.* Thus, in the following, we use *τi→j* to quantify the resting-state effective connectivity and use this measure to investigate the patterns of directional information flow between the different regions of the brain.

## 3. Results

### 3.1. Juggling

**Figure 2:**
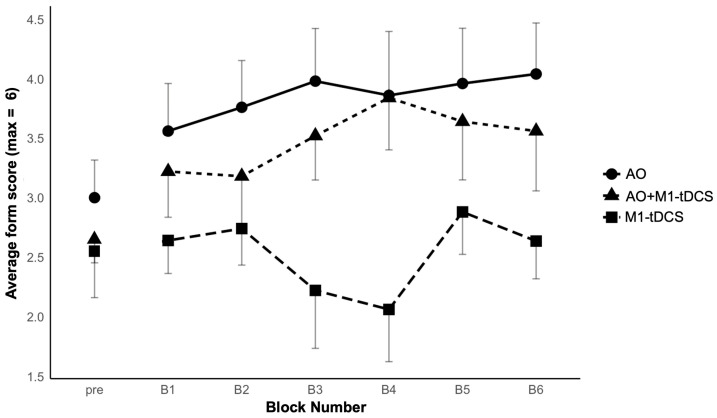
Mean total juggling form scores (and between-participant SEs) for each of the three groups across a 2-trial pre-test and six, 5-trial blocks of post-intervention practice.

Figure 2 shows the group averaged juggling scores for the 2-trial pre-test and across six, 5-trial blocks of post-intervention practice. There was improvement in the two observation groups following observational practice, but not following tDCS alone. These observations were confirmed statistically. First, there were no significant differences between the groups in the pre-test, as confirmed by a one-way ANOVA and two pre-planned between-groups contrasts comparing (i) the two observation groups to the tDCS only group (*p* = 0.43) and (ii) the two observation groups to each other (*p* = 0.48). Second, analysis of the acquisition data following the intervention resulted in significant group differences between the two observation groups and the tDCS only group (*p* = 0.044; diff = −1.02; 95% CI = −2.02 to −0.003), but not between the AO and AO+M1-tDCS groups (*p* = 0.52; diff = −0.37; 95% CI = −1.52 to 0.79). Third, there was a significant block effect (with Greenhouse Geisser adjustment due to sphericity violation), F(3.77, 101.75) = 2.67, *p* = 0.039, *ƞ_p_^2^* = 0.09, due to a general increase in form scores with practice, but Block did not interact with Group, F(7.54, 101.75) = 1.76, *p* = 0.074, *ƞ_p_^2^* = 0.12.

### 3.2. Qualitative Comparison of Effective Connectivity Patterns

Based on analysis of the EEG data, two individuals from the M1-tDCS group and one participant from the AO+M1-tDCS group were excluded from analysis due to excessive EEG noise.

**Figure 3:**
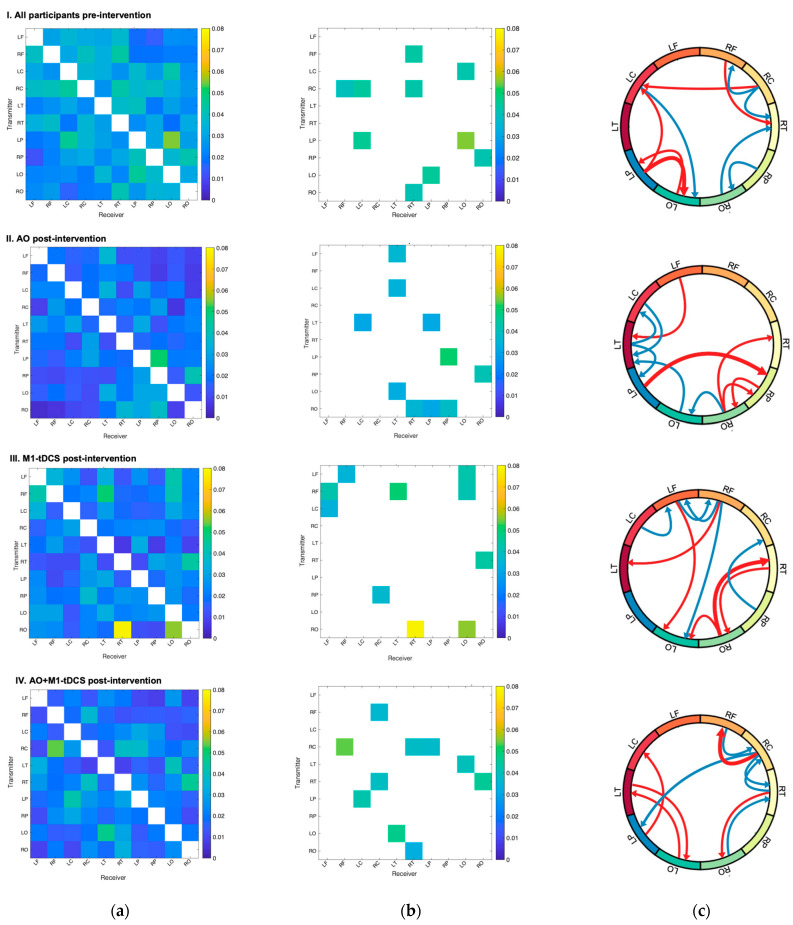
Causal connectivity analysis of EEG source data. Schematics (**I**–**IV**) display from top to bottom: all participants pre-intervention (*N* = 27), AO post-intervention (*n* = 10), M1-tDCS post-intervention (*n* = 8), and AO+M1-tDCS post-intervention (*n* = 9). Labels are listed in Table 1. (**a**) Maps of mean of absolute normalized information flow rate |*τi*→*j*|. Transmitting regions listed on the *y*-axis and receiving regions listed on the *x*-axis. (**b**) Maps of the top 10 mean |*τi*→*j*| values. (**c**) Qualitative summary illustration of the spatial distribution of the directional connections for all groups pre-intervention and each group post-intervention. The Illustration displays the top 10 connections based on the average value of |*τi*→*j*|. The five most important connections are shown in red. The next five top connections (i.e., 6–10) are shown in blue.

Figure 3a shows maps of the mean of absolute normalized information flow rate |*τi*→*j*| with transmitting regions on the *y*-axis and receiving regions on the *x*-axis, and in 3B, maps of the top 10 mean |*τi*→*j*| values are shown to help simplify between-group comparisons. The source region labels are defined in Table 1. The top 10 |*τi*→*j*| values in the AO group ranged from 0.031 to 0.051, the M1-tDCS group ranged from 0.034 to 0.078, and the AO+ M1-tDCS group ranged from 0.032 to 0.053. The top 10 |*τi*→*j*| values in the pre-intervention baseline ranged from 0.040 to 0.055. The mean absolute normalized information flow rates are highest in the post-intervention M1-tDCS group compared to both observation groups as well as the pre-intervention baseline.

Differences in the spatial distribution of normalized information flow rates |*τi*→*j*| pre-intervention and in the three groups post-intervention were seen (see Figure 3b). In the AO only group, the strongest information exchange was from (L) parietal to the (R) parietal region, as well as strong bidirectional information exchange between (R) parietal and (R) occipital regions. In addition, there was an extensive network of activity involving the (L) frontal regions and bidirectional information exchange between central, temporal and occipital regions that was (L) lateralized. In contrast, in the M1-tDCS only group, information exchange was characterized by bilateral long-range connections, with the strongest information exchange from the (R) occipital region to the (R) temporal and (L) occipital regions. In addition, there were extensive long-range connections from (bi) frontal regions to (L) occipital regions and inter-hemispheric, bidirectional information transfer between the (L) and (R) frontal regions that were notably absent in the AO group. In the combined AO+M1-tDCS group, there was a distinct difference in information flow from the previous two groups with strong bidirectional, symmetric information exchange in both hemispheres between the (R) occipital and (R) temporal regions and (L) occipital and (L) temporal regions. In addition, it was the only group that was characterized by information exchange between the (R) frontal and central regions.

A qualitative illustration of the pattern of flow rates for the top 10 mean values is shown in Figure 3c (ranked based on the average value of *|τi→j|*). After observational practice, there is more involvement from the left frontal and parietal regions, as compared to M1-tDCS, and more transmission of information between the two hemispheres.

### 3.3. Statistical Comparison of Effective Connectivity Patterns

**Figure 4:**
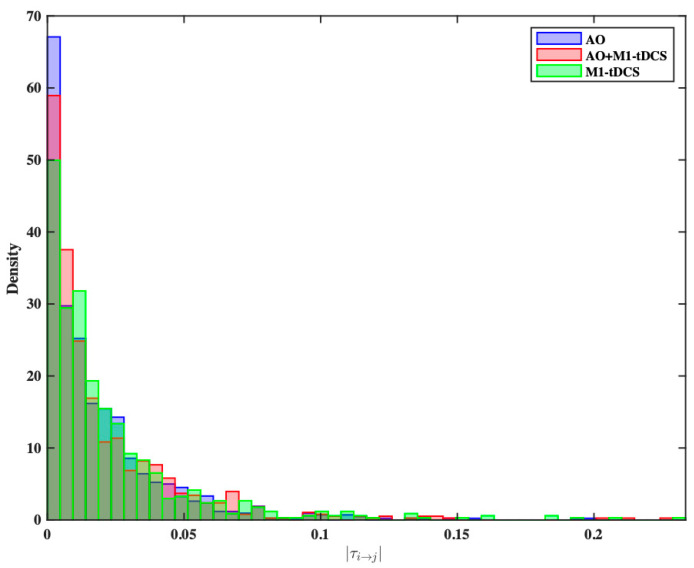
Probability density histograms of the |*τi→j*| values for the M1-tDCS (blue), the AO (rose) group and the AO+MI-tDCS group (green).

We performed a statistical comparison of the normalized information flow rates for each group to determine if the information flow rate distributions were different. To do this, we calculated *|τi→j|* values across participants for each of the 90 unique pairs derived from 10 source locations. This causal connectivity analysis resulted in three distinct statistical distributions, as shown in Figure 4. To evaluate differences in these three distributions, we used the Kolmogorov–Smirnov (KS) test. After adjusting for multiple comparisons using the Bonferroni correction, *p*-values < 0.0167 were considered significant. There were significant differences in the *|τi→j|* distributions between the AO and M1-tDCS groups (*p* = 0.0002), as well as between the AO+M1-tDCS and M1-tDCS groups (*p* = 0.008). There was no significant difference between the AO and AO+M1-tDCS distributions (*p* = 0.335). To further compare the probability distributions of *|τi→j|* values, we used the non-parametric Kruskal–Wallis (KW) test. Based on the KW test, we concluded that the three groups did not come from the same distribution (*p* = 0.0144). Subsequent post hoc analysis using the Dunn-Bonferroni test supported the initial KS test findings, indicating a statistically significant difference between AO and M1-tDCS (Q value = 3.432, critical Q value = 2.388). However, when comparing M1-tDCS to AO+M1-tDCS (q value = 1.556) and AO+M1-tDCS to AO (q value = 1.876), showing no significant distributional differences between these pairs.

## 4. Discussion

In this study, we investigated effective connectivity of resting-state EEG source data underlying skill acquisition of a complex novel bimanual task as a function of two different types of stimulation: direct motor stimulation via M1-tDCS or indirect “passive” stimulation via action observation (AO). We studied both the independent and combined effects of these direct and indirect activation methods. Overall, our data indicate that each intervention activated the brain through distinct network dynamics. M1-tDCS induced bi-directional changes in both local and more widespread bilateral brain regions. AO induced targeted changes in motor and non-motor regions that were associated with the action-perception network, with a dominance of connections in the left hemisphere with (L) frontal and (L) parietal regions driving the brain activity. Different to the independent effects of either type of “stimulation,” AO+M1-tDCS induced strong bidirectional information exchange in both hemispheres in posterior regions of the brain. In addition, it was the only condition that was characterized by strong information exchange in the right hemisphere between the frontal and central regions. Notably, there were significant benefits in measures of juggling accuracy for the two observation groups when compared to the tDCS only group. Combining AO with M1-tDCS also did not improve juggling accurary compared to AO alone.

Contrary to our prediction related to AO, LP was the driver of information flow to RP. Strong bidirectional information transfer was also observed from RO and RP. It is well established that the parietal cortex has a central role in representing and interpreting actions [28,29], with activity in the parietal regions occurring very early following the onset of movement observation [6]. Furthermore, Urgen and Oban [30] have recently proposed that the parietal cortex has a unique role in coding the immediate goal of the observable action. While these previous studies have shown the strongest areas of activation associated with AO, we know little about the primary drivers of this activity. Our data show that left parietal region plays a critical role in driving information flow to the right parietal and that right parietal and occipital regions play a critical role not only in visual-perceptual processing, but also in contributing to a strong network involved in processing complex movements during action observation.

Among the top five connections in the AO group, LF (left frontal) also had strong information transfer to LT. Of note is that LT is a significant node within this network, with bi-directional information exchange occurring from frontal, motor, parietal and occipital regions. We have previously shown that during the observation of a right-handed, goal-directed reaching movement in a live model, the first brain area to be activated was the right temporal region [6], followed by activity in the sensorimotor and parietal regions. These activations suggest that this discrimination between self and other are mediated by early interactions between the temporal regions and the sensorimotor regions, serving as an essential link between perception and action. It has been suggested that the temporal cortex interacts with premotor and parietal cortex particularly during imitation, by integrating visual input with reafferent copies of the imitated action. Recently, Pitcher and Ungerleider [31] proposed a visual pathway from the visual cortex to the superior temporal sulcus (STS) to understand and interpret complex actions. While EEG does not have the spatial resolution for the fine-grained analysis of specific brain regions, our findings from information flow provide further evidence of the important role that the LT region plays in coding and integrating information from observation of a novel, complex task.

Our second prediction was based on past research, showing that the effects of tDCS are not simply limited to regional site-specific effects but rather have widespread effects across the brain [19,32]. Our results add to this literature by providing new information about the direction and strength of these distributed effects. Effective connectivity following direct stimulation of M1 was characterized by strong bilateral information exchange between the (R) occipital region and the (R) temporal regions. More aligned with our prediction, there was strong information exchange from both (L) and (R) frontal regions to (L) occipital and (L) temporal regions, respectively, as well as short-range, inter-hemispheric, bidirectional information transfer between the (L) and (R) motor regions. These results reveal that tDCS not only extends beyond the site of stimulation as reported previously, but that the strongest bidirectional information exchange occurs between right occipital and right temporal regions as well as long-range information flow from left frontal to left occipital regions. In addition, there is evidence of bidirectional, inter-hemispheric connections between left and right frontal regions that are absent in the AO condition. These results indicate that effective connectivity following M1-tDCS has a distinct pattern of activation that involves strong occipital-temporal information exchange and that left M1 stimulation induces motor activation across both hemispheres.

Given the lack of previous research combining AO+M1-tDCS on skill acquisition of a novel task, our third prediction relied on assumptions from the integration of the effects of both modalities on effective connectivity. In part, the pattern we observed did match our prediction, revealing some elements from each condition. For example, we observed strong bidirectional information flow in the posterior regions of the brain, bilaterally, likely related to areas responsible for perception-action coupling via the visual system as discussed above. However, the strong bidirectional information exchange between the (R) frontal and (R) central was unanticipated given the patterns in the previous single conditions.

Using functional connectivity analysis, it has been shown that in the early stages of novel bimanual motor sequence learning there is strong coupling between the frontal and motor networks [33]. This coupling is thought to be related to the ability of the primary motor cortex to reorganize during bimanual learning [33,34]. As such, we hypothesized that the combined stimulation would facilitate this reorganization in the early stages of motor learning, although here we did not find advantages for actual performance from combining AO with tDCS.

These observations not only open new questions for further research but also provide some suggestive insights for motor rehabilitation. It has recently been proposed that top-down and bottom-up stimulation techniques (i.e., peripheral nerve stimulation), combined with AO, could enhance the traditional treatment approaches for stroke rehabilitation [35]. This proposal is based on a review of evidence suggesting that long-term potentiation-like (LTP) plasticity may be the underlying mechanism of acquiring novel motor skills via AO. The underlying mechanism for tDCS is also related to LTP-plasticity, thus combining these two modalities could have increased benefits in a rehabilitation context particularly for those patients who have more severe impairments. However, although we have evidence that at the brain level, combining AO with tDCS elicits short-term changes in functional connectivity, over and above what is seen from either type of stimulation alone, we did not show behavioural effects associated with tDCS; in isolation or in combination with AO. Rehabilitation interventions generally include some elements of AO in the earliest stages of motor re-learning, but often, these approaches have a sequential progression. It is possible that during the early stages of motor rehabilitation, targeted tDCS may increase the ‘readiness’ of the brain following injury and may prepare the brain for more targeted multi-modal intervention. These new and innovative directions for motor rehabilitation require further study.

This study is not without limitations. Differences in motor coordination, sport exposure and experience may well contribute to an individual’s response to AO/M1-tDCS interventions. The small sample size of this study is also more easily influenced by individual differences between intervention groups. Future studies that involve greater sample sizes are needed. We also did not include a sham tDCS group in our study to distinguish placebo effects related to stimulation. However, this was not necessary here as there were no behavioural benefits associated with tDCS alone and we already had comparison groups where distinct effects of tDCS could be gauged from comparisons of AO alone to AO+M1-tDCS. There are also potential generalization issues associated with the task we chose (i.e., 2-handed two-ball juggling) and measures necessary to infer improvements. We chose this task because it better represents skill learning distinct from simple memory advantages associated with watching repeated sequences for example. However, the potential complexity involved in coordinating a 2-handed action that requires physical practice could lessen the impact of our intervention conditions. Further studies with a larger range of skills and potentially more diverse populations are needed to determine the generalizability of these effects.

## 5. Conclusions

Overall, these data indicate that both AO and tDCS serve as tools for neural enhancement and that they do so through different and distinct cortical mechanisms. While all three interventions demonstrated a clear impact on the plasticity of resting-state effective connectivity, there was no evidence that tDCS enhanced motor skill acquisition, as evidenced through a lack of improvement in juggling accuracy compared to AO. The AO groups outperformed the tDCS only group and there were no additional benefits in accuracy associated with combining of AO with tDCS. These results suggest that AO stimulates the brain in a more task-specific and goal-directed manner within the traditional mirror neuron network, as well as broader visual-perceptual processes. Conversely, tDCS alone activated the brain in a more generalized manner, in preparation or readiness for a variety of different goals and behaviours. This research, where we have combined tDCS with effective connectivity brain analysis, offers a novel framework and methodology to understand the network related changes in the brain as a result of indirect (AO) and direct (tDCS) brain stimulation.

## Figures and Tables

**Table 1:** List of 10 brain regions and associated abbreviations used for the EEG source reconstruction.

Label	Source Region
LF	Frontal, left hemisphere
RF	Frontal, right hemisphere
LC	Central, left hemisphere
RC	Central, right hemisphere
LT	Temporal, left hemisphere
RT	Temporal, right hemisphere
LP	Parietal, left hemisphere
RP	Parietal, right hemisphere
LO	Occipital, left hemisphere
RO	Occipital, right hemisphere

## Data Availability

Date may be available by contacting the authors (N.J.H. and N.V.-B.).The data are not publicly available due to containing information that could compromise the privacy of research participants.
